# Supplementary material for: PHC1 maintains pluripotency by organizing genome-wide chromatin interactions of the Nanog locus
Source: Nat Commun. 2021 May 14;12:2829. doi: 10.1038/s41467-021-22871-0 (PMC8121881; doi:10.1038/s41467-021-22871-0)
Supplement: Supplementary file 1 — Supplementary Information [file 41467_2021_22871_MOESM1_ESM.pdf]

Fig. S1

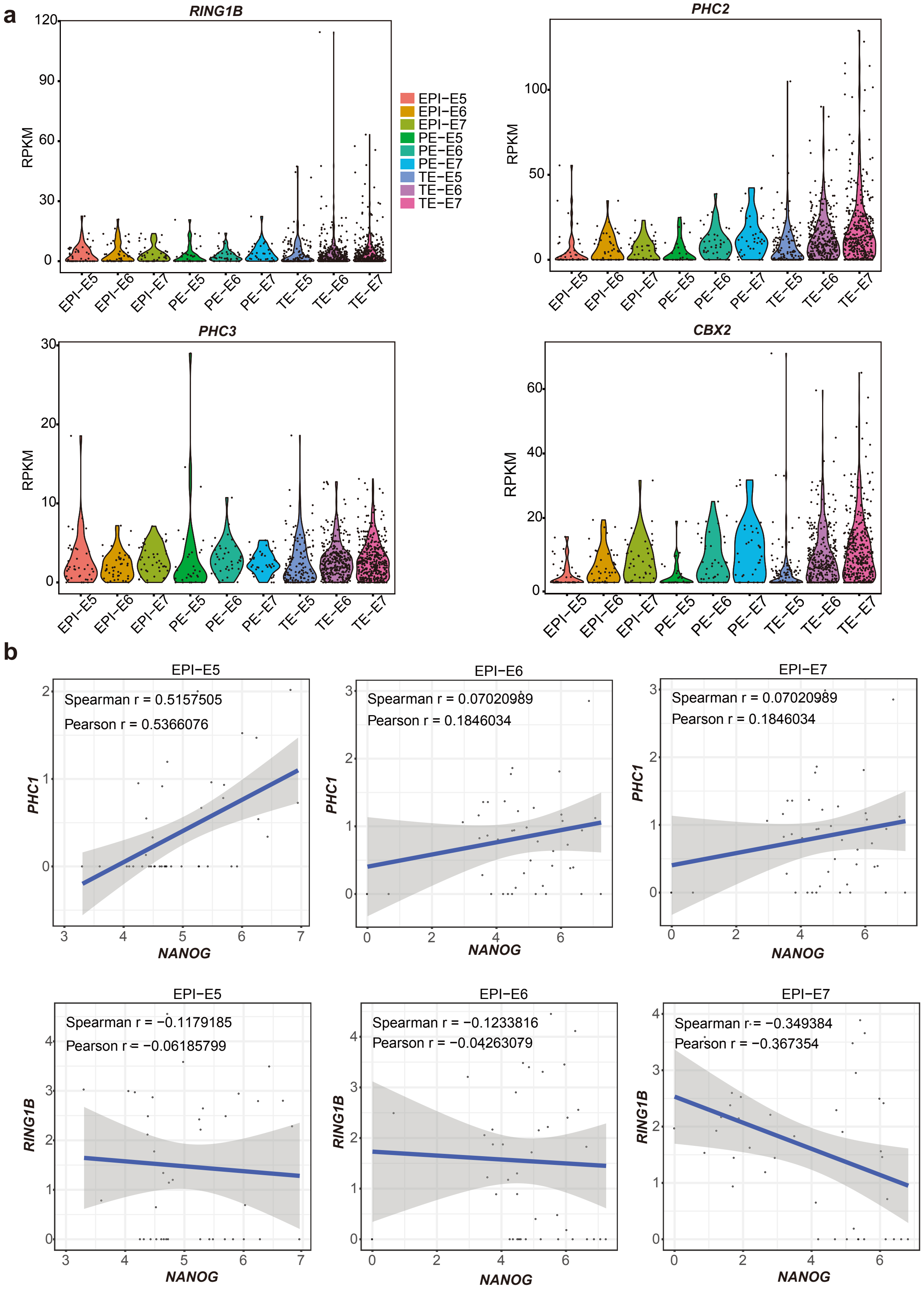
a
RING1B
PHC2
PHC3
CBX2
RPKM
EPI−E5
EPI−E6
EPI−E7
PE−E5
PE−E6
PE−E7
TE−E5
TE−E6
TE−E7
b
EPI−E5
Spearman r = 0.5157505
Pearson r = 0.5366076
EPI−E6
Spearman r = 0.07020989
Pearson r = 0.1846034
EPI−E7
Spearman r = 0.07020989
Pearson r = 0.1846034
PHC1
NANOG
EPI−E5
Spearman r = −0.1179185
Pearson r = −0.06185799
EPI−E6
Spearman r = −0.1233816
Pearson r = −0.04263079
EPI−E7
Spearman r = −0.349384
Pearson r = −0.367354
RING1B
NANOG

Fig. S1: Analysis and correlation of PRC1 genes with *NANOG* expression in early human embryos (Related to Fig. 1). a, Analysis of the published single-cell RNA-seq data of early human embryos (E5-7) showing expression of *RING1B, PHC2, PHC3* and *CBX2* in epiblast (EPI), primitive endoderm (PE) and trophectoderm (TE)[32]. **b**, Correlation of *NANOG* with *PHC1* and *RING1B* expressions in EPI lineage at E5. Two-sided t test was used with 95% confidence intervals.

Fig. S2

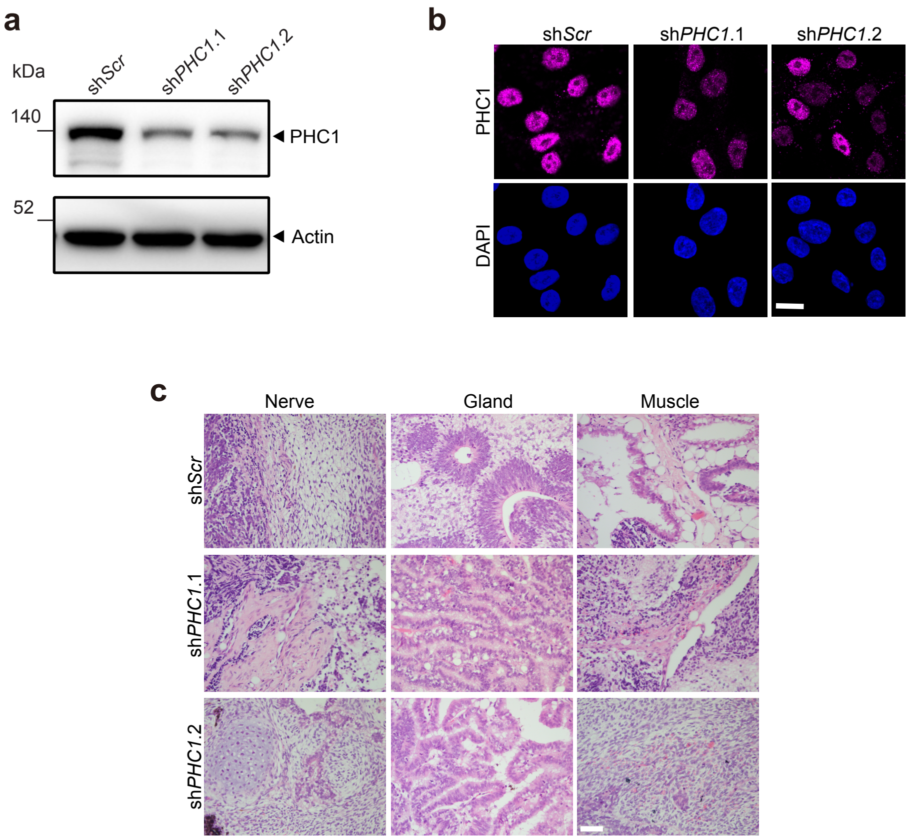

Fig. S2: *PHC1* knockdown reduces teratoma formation capacity of hESCs in NOD/SCID mice (Related to Fig. 2). **a,** WB analysis of PHC1 expression level in the sh*Scr* and sh*PHC1*-infected hESCs. **b,** Immunofluorescent staining of PHC1 in the sh*Scr* and sh*PHC1*-infected hESCs. Scale bars, 20μm. **c,** Histological analysis of the tumors formed by sh*Scr* and sh*PHC1*-infected hESCs showing neural, gland and muscle tissues representative of ectoderm, endoderm and mesoderm respectively. Scale bars, 20μm. Source data are provided as a Source Data file.

Fig. S3

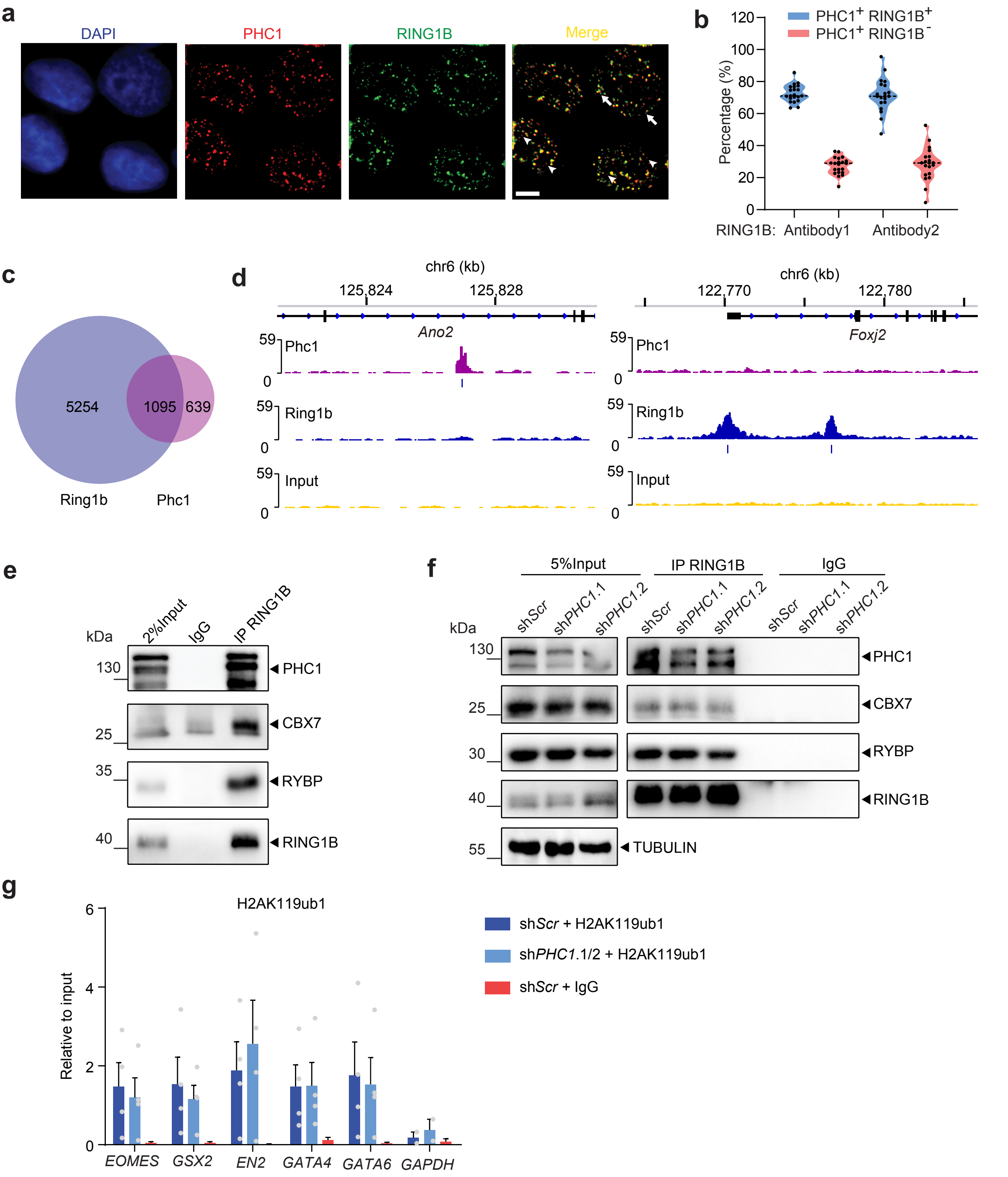

Fig. S3: Suppression of *PHC1* impairs pluripotency of hESCs without affecting PRC1 assembly and occupancy of target genes (Related to Fig. 2). a, Super-resolution microscopic imaging analysis of PHC1 and RING1B co-immunostaining in hESCs. Arrows showed RING1B green signals without overlapping with PHC1 signals and arrowheads showed single red PHC1 signals without co-localizing with RING1B green signals. Scale bars, 5µm. **b,** Quantification of percentages of $PHC1^{+}$ $RING1B^{+}$ and $PHC1^{+}RING1B^{-}$ foci in hESCs immunostained with PHC1 and two independent RING1B antibodies, respectively. 41 cells were counted. **c,** Venny diagram analysis of genes bound by Phc1 and Ring1b in mESCs[16]. **d,** IGV images showing *Ano2* and *Foxj2* genes with non-overlapping Phc1 and Ring1b binding peaks in mESCs, respectively. **e,** RING1B IP followed by WB analysis of PHC1, CBX7 and RYBP using extracts from hESCs. **f,** RING1B IP was performed with the sh*Scr*- and sh*PHC1*-infected hESCs extracts followed by immunoblotting of CBX7, RYBP, and RING1B. IgG IP was used as the negative Ctrl. **g,** ChIP-PCR analysis of H2AK119ub1 density at target genes including *EOMES, GSX2, EN2, GATA4 and GATA6,* and *GAPDH*, a non-target control, in the sh*Scr*- and sh*PHC1*-infected hESCs. sh*Scr* + IgG was used as the negative control. Data are the mean ± s.e.m. of n=4 independent experiments. Source data are provided as a Source Data file.

Fig. S4

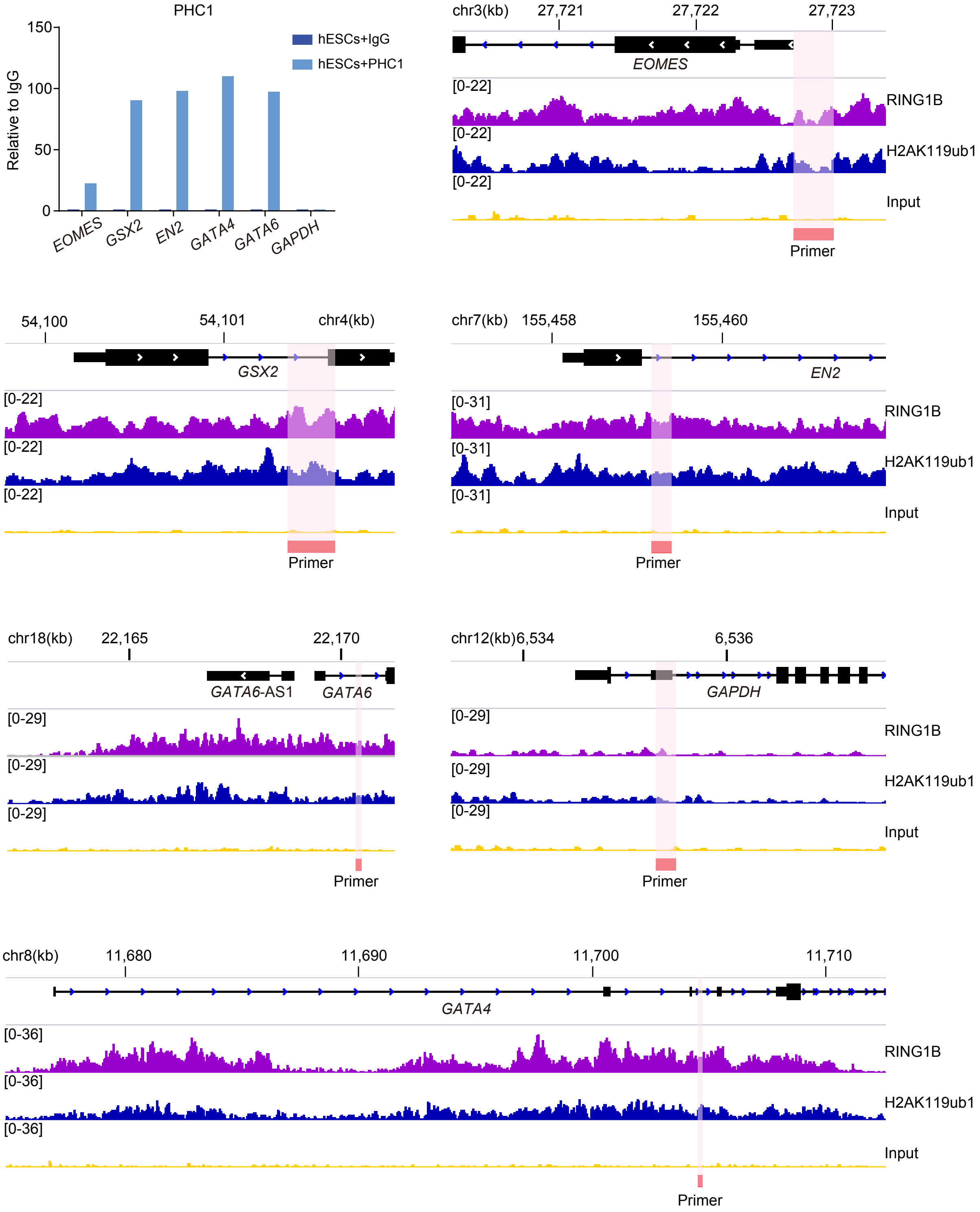

Fig. S4: Binding of PHC1, RING1B and H2AK119ub1 to developmental genes in hESCs. ChIP-PCR analysis of PHC1 binding at target genes including *EOMES, GSX2, EN2, GATA4, GATA6,* and *GAPDH*, a non-target control, in wild type hESCs. IgG was used as the negative control. IGV images of RING1B and H2AK119ub1 density at these genes were also shown. Primers used for H2AK119ub1 ChIP-PCR analysis on these genes in Fig.S3g were indicated. Source data are provided as a Source Data file.

Fig. S5

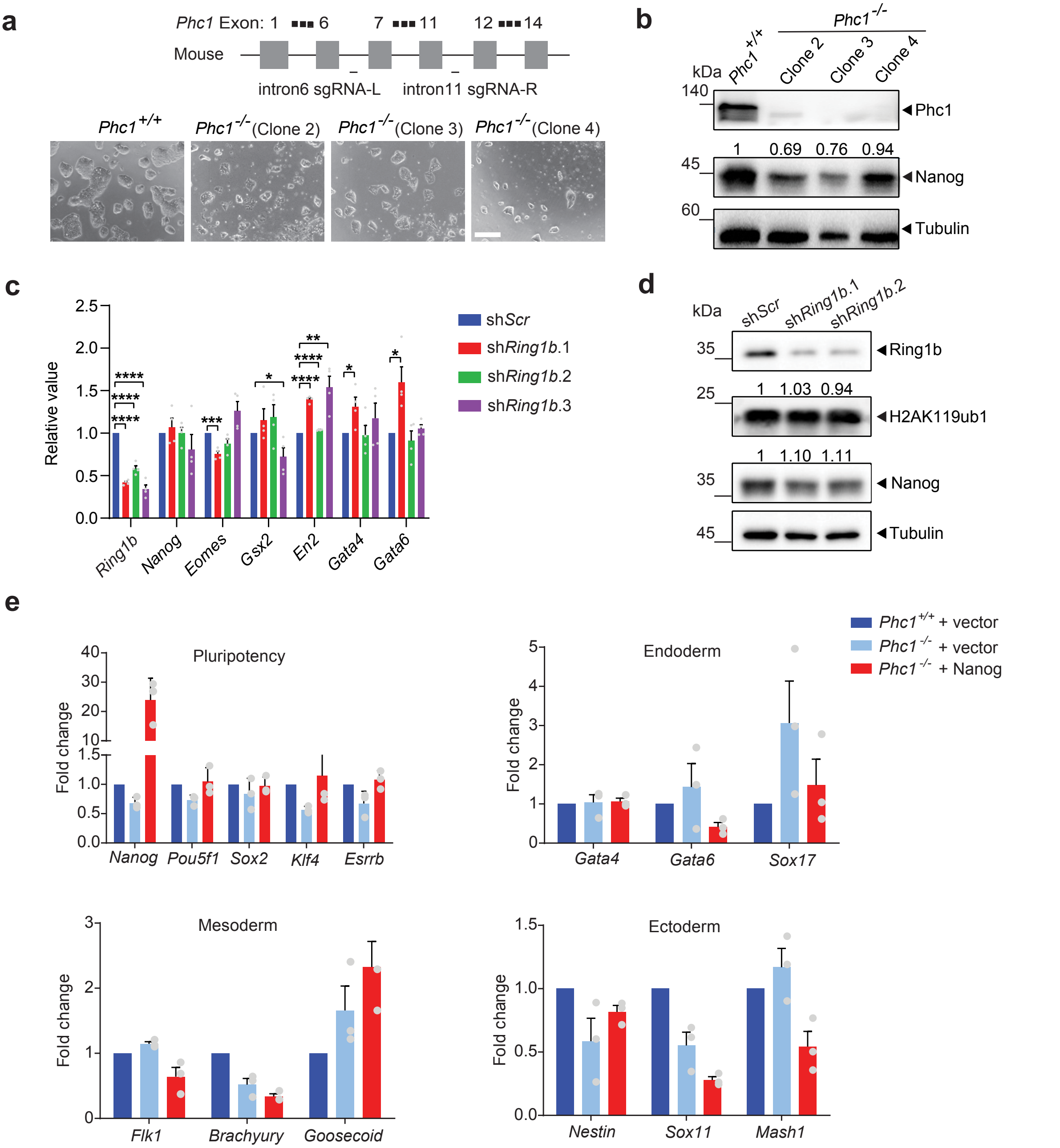

Fig. S5: Overexpression of Nanog in *Phc1*$^{-/-}$ mESCs partly rescued gene expressions (Related to Fig. 3). a, Designing a pair of sgRNAs targeting the 6th and 11th introns of mouse *Phc1* gene, respectively, and the morphology of the *Phc1*$^{+/+}$ and *Phc1*$^{-/-}$ mESCs (clones 2-4). Scale bars, 600μm. **b,** WB analysis of Phc1, Nanog, and Actin protein in the *Phc1*$^{+/+}$ and *Phc1*$^{-/-}$ mESCs (clones 2-4). **c,** qPCR analysis of *Ring1b, Nanog, Eomes, Gsx2, En2, Gata4* and *Gata6* in the sh*Scr*- and sh*Ring1b*-infected mESCs. Data are the mean ± s.e.m. of n=4 independent experiments. Two-tailed unpaired t-tests were used (p<0.0001 for *Ring1b* in all groups; p= 0.0003 for *Eomes*; p=0.0388 for *Gsx2*; p<0.0001 for *En2* in sh*Scr* vs. sh*Ring1b*.1; p<0.0001 for *En2* in sh*Scr* vs. sh*Ring1b*.2; p=0.0062 for *En2* in sh*Scr* vs. sh*Ring1b*.3; p=0.0317 for *Gata4*; ***p=0.0164 for *Gata6*). **d,** Immunoblotting of Ring1b, H2AK119ub1, Nanog and Tubulin protein in the sh*Scr*- and sh*Ringlb*-infected mESCs. **e,** qPCR analysis of genes representative of pluripotency, endoderm, mesoderm and ectoderm germ layers for the *Phc1*$^{+/+}$ + vector, *Phc1*$^{-/-}$+ vector, and *Phc1*$^{-/-}$ + Nanog mESCs. Data are mean ± s.e.m of 3 independent experiments. Source data are provided as a Source Data file.

Fig. S6

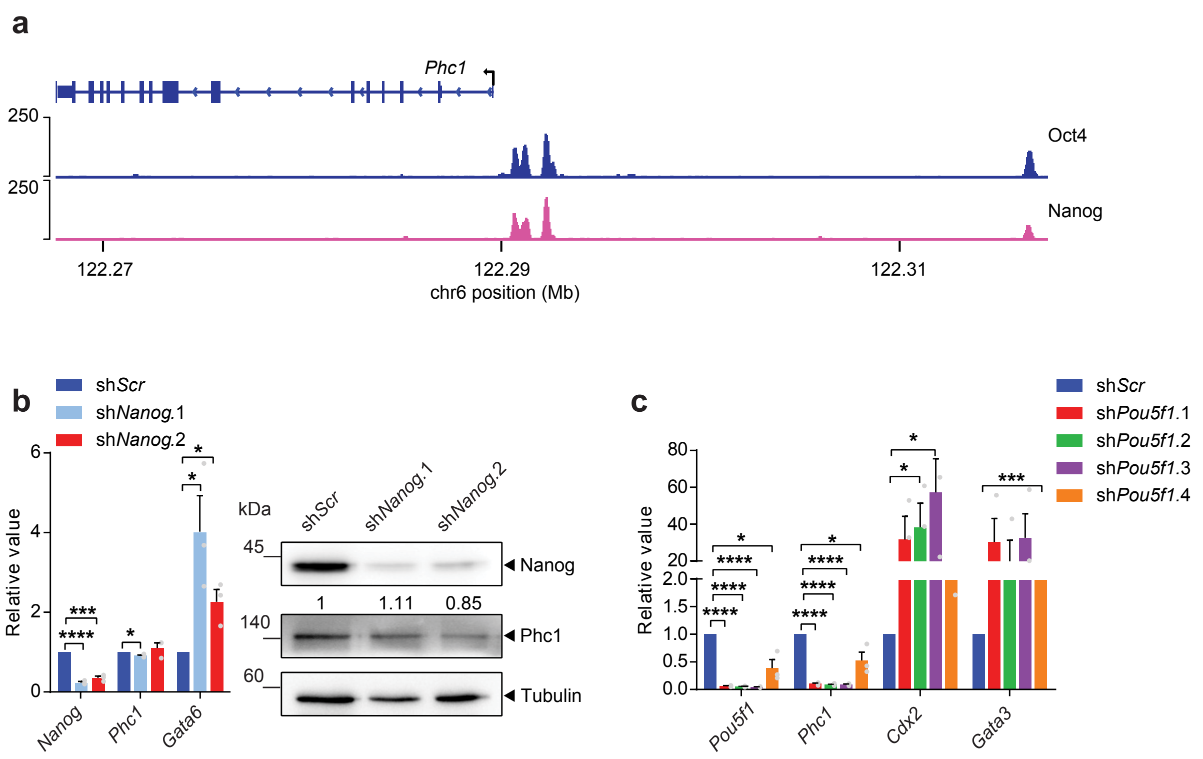

Fig. S6: Oct4 is the upstream regulator of *Phc1* in mESCs (Related to Fig. 3). a, The IGV image showing ChIP-seq analysis of Oct4 and Nanog binding profiles at the *Phc1* promoter region[8] . **b,** qPCR analysis of *Nanog, Phc1* and *Gata6* and immunoblotting in the sh*Scr* and sh*Nanog*-infected mESCs. Data are the mean ± s.e.m of n=3 independent experiments. Two-tailed unpaired t-tests were used (*****p*<0.0001 and ****p*=0.0002 for *Nanog*; **p*=0.0161 for *Phc1*; **p*=0.0290 for *Gata6* in sh*Scr* vs. sh*Nanog*.1; **p*=0.0154 for *Gata6* in sh*Scr* vs. sh*Nanog*.2). **c,** qPCR analysis of *Pou5f1, Phc1, Cdx2 and Gata3* in the sh*Scr*- and sh*Pou5f1*-infected mESCs. Data are the mean ± s.e.m of n=3 independent experiments. Two-tailed unpaired t-tests were used (*****p*<0.0001 for *Pou5f1* in sh*Scr* vs. sh*Pou5f1*.1, sh*Pou5f1*.2 and sh*Pou5f1*.3; **p*=0.0161 for *Pou5f1*; *****p*<0.0001 for *Phc1* in sh*Scr* vs. sh*Pou5f1*.1, sh*Pou5f1*.2 and sh*Pou5f1*.3; **p*=0.0357 for *Phc1*; **p*=0.0460 for *Cdx2* in sh*Scr* vs. sh*Pou5f1*.2; **p*=0.0370 for *Cdx2* in sh*Scr* vs. sh*Pou5f1*.3; ****p*=0.0005 for *Gata3*). Source data are provided as a Source Data file.

Fig. S7

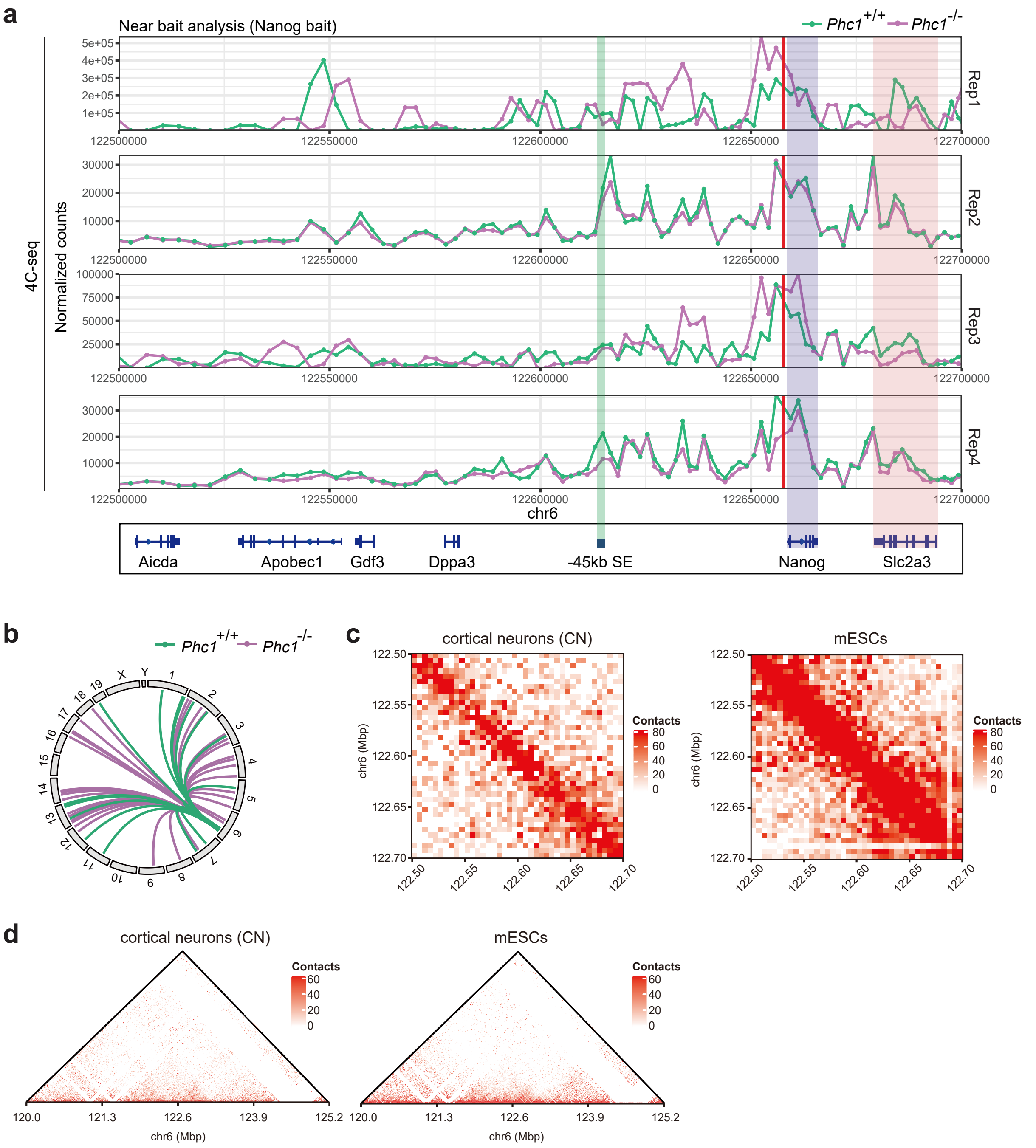

a
Near bait analysis (Nanog bait)
Phc1+/+
Phc1-/-
4C-seq
Normalized counts
Rep1
Rep2
Rep3
Rep4
122500000
122550000
122600000
122650000
122700000
chr6
Aicda
Apobec1
Gdf3
Dppa3
-45kb SE
Nanog
Slc2a3
b
c
cortical neurons (CN)
mESCs
Contacts
chr6 (Mbp)
d
cortical neurons (CN)
mESCs
Contacts
chr6 (Mbp)

Fig. S7: *Phc1* deficiency influences genomic interactions of the *Nanog* locus (Related to Fig. 5). a, Analysis of four replicates of 4C-seq datasets showing interaction profiles of genomic regions with the *Nanog* promoter (anchor) in chr6 of *Phc1*$^{+/+}$ (green line) and *Phc1*$^{-/-}$ (purple line) mESCs. X and Y axes indicate chromosome coordinates around the *Nanog* locus and the normalized interaction intensity, respectively. Chr6: 122500000-122700000 of mm9 genome assembly was shown. The peak interaction was normalized to the highest interaction fragment (chr6: 122654169 fragment) in the control sample of each replicate. **b,** Circos plot of inter- and intra-chromosomal interactions with the *Nanog* promoter (anchor) in chr6: 122500000-122700000 of mm9 genome assembly in *Phc1*$^{+/+}$ (green line) and *Phc1*$^{-/-}$ (purple line) mESCs. The green and purple colors refer to interactions between the *Nanog* promoter (anchor) with other chromosomes and regions within chr6 in *Phc1*$^{+/+}$ and *Phc1*$^{-/-}$ mESCs, respectively (n=4). **c,** Hi-C interaction matrices of mouse CN and mESCs in chr6: 122500000-122700000 of mm9 genome, respectively[58]. **d,** Hi-C interaction matrices of mouse CN and mESCs in an extended region in chr6:120049982-125249982 of mm9 genome assembly, respectively[58].

Fig. S8

Gating strategies

1.Blank (E14 mESCs):   Ungated ➤ C ➤ A ➤ B

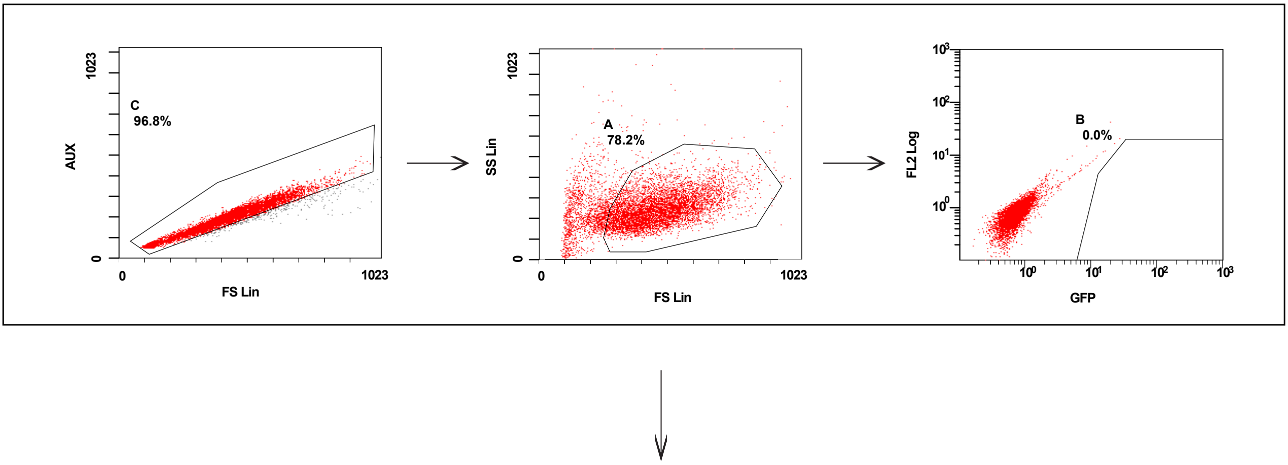

2.Sample cells (mESCs line carrying the *Nanog*-GFP reporter):

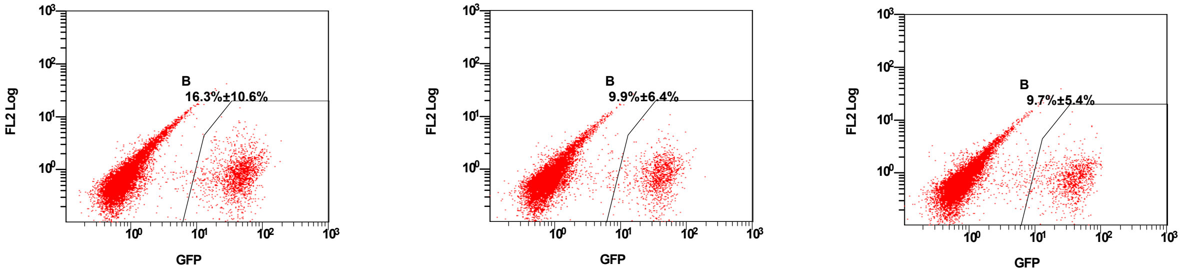

sh*Scr*   sh*Phc1*.1   sh*Nanog*.2

Fig. S8: Gating strategies used for analyzing the selected cell populations (Related to Fig.3d). Gating strategy to analyze GFP+ cells from mESCs line carrying the *Nanog*-GFP reporter infected with indicated lentiviruses. E14 mESCs were used as blank.

Supplementary Table 1-PCR Primers

QPCR Primers:

Gene name	Sequence (5'-3')	Sequence (5'-3')
RING1A (human)	CTACGGAGCGGGAACAAGG	AAGCACTCGGTCTTGATGGG
RING1B (human)	AGCGGCCCCTGTTGTATTTC	CCTGAGACATTGCGGCTCC
CBX2 (human)	GCAGCATGGAGGAGCTGAG	GCTCCCAGCTGTTATGTTTGG
CBX4 (human)	CCTTCCAGAACAGGGAACGG	TCAGGACATTGGAACGACGG
CBX6 (human)	TCGAACAAAAGGAGAGGGAGC	CGGCTTGACAGAGAAATGCAC
CBX7 (human)	TGGCCTACGAGGAGAAGGAG	CACGTCAGGGAGAAGCAGAG
CBX8 (human)	CAACATGGAGCTTTCAGCGG	ATTCCATGCGTCCTTTCCGT
PCGF2 (human)	AGTTTCTCCGCAACAAGAT	AGATGTAGGCGATGTCCA
PCGF4 (human)	GCTGGTTGCCCATTGACAG	AAAAATCCCGGAAAGAGCAGC
PHC1 (human)	CTCGATCAATCTGGCCACCA	CAGCTGTGGCCGTAGATACAT
PHC2 (human)	GACAGGGCAGGGCATTGTTC	CGTCCCACCGGGAAAGG
PHC3 (human)	TGCGCAGCCCATGTTAGT	GAATTACCTGTACAGCATGTCG
NANOG (human)	ACAGAAATACCTCAGCCTCCA	CTGCGTCACACCATTGCTAT
POU5F1 (human)	CCTCACTTCACTGCACTGTA	CAGGTTTTCTTTCCCTAGCT
EZH1 (human)	AGAATGGAAGAAGCTTCGTGTC	GGAACCAATGCAACTGTGTT
EZH2 (human)	ACGATGATGATGATGGAGACGA	TGCTGTGCCCTTATCTGGAA
EED (human)	TGGTGGCAATATTTGGAGGC	CGCCCAAGAATAGTCACATTAGA
SUZ12 (human)	GTGTTACCGGTGAAGAAGCC	TGGTGCTATGAGATTCCGAGT
Nanog (mouse)	AAAGGATGAAGTGCAAGCGG	CTTCCAGATGCGTTCACCAG
Pou5f1 (mouse)	CCTTGCAGCTCAGCCTTAAG	CCTCACACGGTTCTCAATGC
Sox2 (mouse)	GCACATGAACGGCTGGAG	GTCGTAGCGGTGCATCGG
Klf4 (mouse)	AAAAGAACAGCCACCCACAC	GGTAGTGCCTGGTCAGTTCA
Esrrb (mouse)	TGAGATCACCAAACGGAGGC	GTTCAGGTAGGGGCTGTTCT
Tbx3 (mouse)	CATCCTCTCCTGCTGTCTCC	GTGCTCCTCCTTGCTCTCA
Gata4 (mouse)	CCCCTTCGACAGCCCAGT	GTCCCATCTCGCCTCCAG
Gata6 (mouse)	CAGCAGGACCCTTCGAAAC	CCGTCTCGTCTCCACAGTG
Sox17 (mouse)	GTGTGGGCCAAAGACGAAC	GCTTCTCTGCCAAGGTCAAC
Flk1 (mouse)	ACATCACCGAGAACAAGAACA	AACTGGGGAGAGTAAAGCCT
Brachyury (mouse)	CCCAGACTCGCCCAATTTTG	CTGGTGATCATGCGTTGCG
Goosecoid (mouse)	GAGGAGAAGGTGGAGGTCTG	GTCTTGTTCCACTTCTCGGC
Nestin (mouse)	GAACTCTCGCTTGCAGACAC	GGGGAAGAGAAGGATGTTGG
Sox11 (mouse)	CTGTCGCTGGTGGATAAGGA	CTGCGCCTCTCAATACGTG
Mash1 (mouse)	CCAACTGGTTCTGAGGACCT	CTCCCCATTTGACGTCGTTG
Phc1 (mouse)	CCAAGAAGGCAGAAGCAGAT	TCTGCTGCTGTTGCTGAATC
Cdx2 (mouse)	CTTCAACGTTTGTCCCCAGA	CACGGAGCTAGGATACATGC
Gata3 (mouse)	AAAGAAGGCATCCAGACCCG	TTGAAGGAGCTGCTCTTGGG
Ring1b (mouse)	ACACAACAATCAGCAGGCTC	CTGTGTGTGGATGCGTTACT
Eomes (mouse)	CCAAGACTCAGACCTTCACCT	GTTTTGAACGCCGTACCGAC
Gsx2 (mouse)	CCGCCACTACCTACAACATG	GCTGTCCTCATCCTTTTGCC
En2 (mouse)	CCGCTTGGGTCTACTGCA	AGGCCGCTTGTCCTCTTT

Supplementary Table 2-ChIP-PCR Primers

ChIP-PCR primers:

gene name	Sequence (5'-3')-hg38	location:(5'-3')-hg38
EOMES（human）	TGAGATGACTAATGCGCCAAG	27723013-27722993 (chr3)
	GGCTGTCACTAGCTGCTTTAT	27722719-27722739 (chr3)
GSX2（human）	TCTTTAATCCTGTGACTTCGCA	54101357-54101378 (chr4)
	CATCCTCTTGCCATTGGGTAC	54101622-54101602 (chr4)
EN2（human）	CGCACACGCACAAAGACT	155459174-155459191 (chr7)
	GGGAGACATGGGCTAGGG	155459405-155459388 (chr7)
GATA4（human）	GAACGCTTTCAATCCCTGTC	11704560-11704579 (chr8)
	CGACAAGCCTCCGTCTAC	11704728-11704711 (chr8)
GATA6（human）	ACAAGCTCTCCGCATTGC	22170405-22170422 (chr18)
	CGCCGCGTTCTCCTATATT	22170568-22170550 (chr18)
GAPDH（human）	GCAACCGGGAAGGAAATGAATGGG	6535303-6535326 (chr12)
	AGAGGACCTCCATAAACCCACTTC	6535503-6535480 (chr12)

Supplementary Table 3-gRNA and shRNA

target mouse *Phc1* (clone1):	Sequence (5'-3')
sgRNA-L	ACTGCAGCGGCAACCTAATGCGG
sgRNA-R	GGGGGAGTTACTGCAAGGTTAGG
target mouse *Phc1* (clone2,clone3,clone4):	Sequence (5'-3')
sgRNA-L	AAGGTGACTGCCGATTTAAGGGG
sgRNA-R	GGAAATGACATACTAACGTGAGG
target human *PHC1* :	Sequence (5'-3')
sgRNA	CACCACTCCTGTACCCTTTCTGG
PHC1 shRNA target sequence	Sequence (5'-3')
shRNA1	GAGTTTCAAGAAGCCAACTAT
shRNA2	GCCCAGCCAGACACTTATTAT

Ring1b shRNA target sequence	Sequence (5'-3')
shRNA1	TAAGGCCAGACCCGAACTTTG
shRNA2	CCGAGTAACAAACGGACCAAA
shRNA3	CCCTTAGAAGTGGCAACAAAG
Nanog shRNA target sequence	Sequence (5'-3')
shRNA1	GCCAACCTGTACTATGTTTAA
shRNA2	GGAGTATCCCAGCATCCATTG
Pou5f1 shRNA target sequence	Sequence (5'-3')
shRNA1	CGTTCTCTTTGGAAAGGTGTT
shRNA2	CCTACAGCAGATCACTCACAT
shRNA3	GCCGACAACAATGAGAACCTT
shRNA4	GGCTCTCCCATGCATTCAAAC

Supplementary Table 4-antibody

Antibody used in this paper

name	brand	Cat.NO
Anti-Nanog antibody	Cell Signaling	4893(WB)
Anti-Oct4 antibody	Cell Signaling	2750(WB, IF)
Anti-Sox2 antibody	Cell Signaling	23064(WB,IF)
Anti-Nanog antibody	BETHYL	A300-397A(WB)
Anti-Nanog antibody	Cell Signaling	4903(IF)
Anti-PHC1 antibody	Cell Signaling	13768(WB, IP, ChIP)
Anti-PHC1 antibody	Active Motif	39723(IF)
Anti-Oct4 antibody	abcam	ab200834(WB)
Anti-RING1B antibody	Cell Signaling	5694(WB, IP, IF)
Anti-RING1B antibody	abcam	ab181140(WB,IF)
Anti-beta Actin antibody	Cell Signaling	3700(WB)
Anti-beta Tubulin antibody	abcam	ab179513(WB)
Anti-Ubiquityl-Histone H2A (Lys119) antibody	Cell Signaling	8240(WB,ChIP)
Anti-FLAG antibody	MBL	M185-7(WB)
Anti-HA antibody	MBL	M180-7(WB)
Anti-CBX7 antibody	abcam	ab91431(WB)
Anti-RYBP antibody	abcam	ab185971(WB)
Anti Nanog antibody	Cell Signaling	8822(IF, ChIP)
Anti Gata6 antibody	Cell Signaling	5851(IF)
Anti Sox2 antibody	R&D	AF2018(IF)
Control mouse IgG	Sigma	I8765(IP, ChIP)
Control rabbit IgG	Cell Signaling	2729(IP, ChIP)

Supplementary Table 5-4C PCR Primers

4C PCR Primers:

Sequence (5'-3')	Sequence (5'-3')
CGGCTCACTTCCTTCTGACT	CACAGTTAATCCCACCTGCAG

Supplementary Table 7- Datasets of ChIP-seq used in this study

Dataset	Platform	Source	Reference
**H3K27ac**	Illumina Genome Analyzer II	GSE72886 (GSM1874094)	*Kumar et al*., 2016
**Nanog**	Illumina HiSeq 2000	GSE44288 (GSM1082342)	*Whyte et al*., 2013
**Phc1**	Illumina HiSeq 2500	GSE89949 (GSM2393580)	*Kundu et al*, 2017
**H2AUb**	Illumina HiSeq 2500	GSE89949 (GSM2393583)	*Kundu et al*, 2017
**Ring1b**	Illumina HiSeq 2500	GSE89949 (GSM2393579)	*Kundu et al*, 2017
**Oct4**	Illumina HiSeq 2000	GSE44288 (GSM1082340)	*Whyte et al.,* 2013
**RING1B**	Illumina HiSeq 2500	GSE104690 (GSM2805868)	*Wang Z et al.,* 2018
**H2AUb**	Illumina HiSeq 2500	GSE104690 (GSM2805871)	*Wang Z et al.,* 2018
