# Supplementary material for: PHC1 maintains pluripotency by organizing genome-wide chromatin interactions of the Nanog locus
Source: Nat Commun. 2021 May 14;12:2829. doi: 10.1038/s41467-021-22871-0 (PMC8121881; doi:10.1038/s41467-021-22871-0)
Supplement: Supplementary file 3 — Reporting Summary [file 41467_2021_22871_MOESM3_ESM.pdf]

nature research

Corresponding author(s): Junfeng Ji, Feiqiu Wen, Chen Li

Last updated by author(s): 2021-Mar-31

Reporting Summary

Nature Research wishes to improve the reproducibility of the work that we publish. This form provides structure for consistency and transparency in reporting. For further information on Nature Research policies, see our Editorial Policies and the Editorial Policy Checklist.

Statistics

For all statistical analyses, confirm that the following items are present in the figure legend, table legend, main text, or Methods section.

n/a	Confirmed	
☐	☒	The exact sample size (n) for each experimental group/condition, given as a discrete number and unit of measurement
☐	☒	A statement on whether measurements were taken from distinct samples or whether the same sample was measured repeatedly
☐	☒	The statistical test(s) used AND whether they are one- or two-sided<br>*Only common tests should be described solely by name; describe more complex techniques in the Methods section.*
☒	☐	A description of all covariates tested
☐	☒	A description of any assumptions or corrections, such as tests of normality and adjustment for multiple comparisons
☐	☒	A full description of the statistical parameters including central tendency (e.g. means) or other basic estimates (e.g. regression coefficient) AND variation (e.g. standard deviation) or associated estimates of uncertainty (e.g. confidence intervals)
☐	☒	For null hypothesis testing, the test statistic (e.g. F, t, r) with confidence intervals, effect sizes, degrees of freedom and P value noted<br>*Give P values as exact values whenever suitable.*
☒	☐	For Bayesian analysis, information on the choice of priors and Markov chain Monte Carlo settings
☒	☐	For hierarchical and complex designs, identification of the appropriate level for tests and full reporting of outcomes
☐	☒	Estimates of effect sizes (e.g. Cohen's d, Pearson's r), indicating how they were calculated

Our web collection on statistics for biologists contains articles on many of the points above.

Software and code

Policy information about availability of computer code

Data collection	ChemiScope 3300 Mini (CLiNX) with Clinx ChemiCapture (v2.4.10.84) was used for collecting western blot image data. Light Cycler 480 II machine (Roche) with LightCycler 480 (v1.5.1.62) was used for acquiring quantitative real-time PCR data. BD Cytomics FC 500MCL machine with CXP software (v2.3) was used for acquiring flow cytometry data. Olympus BX61 confocal microscope with FV10-ASW (v4.2.3.6) and Nikon SIM microscope with NIS-Elements (v4.30) were used for collecting immunofluorescent super-resolution images. Olympus IX71 microscope with BGIMAGING CellView (v4.10) was used for acquiring regular immunofluorescent images. BGISEQ-500 machine (BGI) and Illumina Novaseq platform was used for collecting RNA-seq data and 4C-seq data, respectively.

Data analysis	All of the statistical analysis were done with GraphPad Prism (v8.0.2). Flow cytometry data were analyzed with FlowJo software (v7.6) or CXP software (v2.3). ImageJ software (v2.1.4.7) was used to quantify the immunofluorescent intensities and the signal intensities of western blot images. Super-resolution microscopic images were analyzed with Imaris software (v9.5). For RNA-seq analysis, reads were aligned to genome assembly using Bowtie2 (v2.2.5). RSEM (v1.2.15) was run to quantify the expression FPKMs of each annotated transcript RefSeq. For 4C-seq analysis 4C data was processed using 4C-ker package (v1.0). 4C-seq reads were trimmed and mapped to genome by Bowtie2 (v2.3.4.1). The counts profiles were used to analyze near cis-, far cis- and trans-interactions by 4C-ker (v1.0). The far cis- and trans- interaction profiles were visualized by ggbio (v1.30.0). For ChIP-seq analysis, data were aligned to genome assembly using Bowtie2 (v2.3.4.2). Peak calling was performed by MACS2 program (v2.1.1.20160309). Each peak was annotated with its nearest gene using the R (v3.4.0) package ChIPseeker (v1.10.3). Genomic annotation and gene coordinates were obtained from a BioConductor package TxDb.Mmusculus.UCSC.mm9.knownGene (v3.2.2). The resulting normalized signal enrichment file in bigWig format was visualized on the Integrative Genomics Viewer (IGV) (v2.4.14). For Hi-C data analysis, the archives were extracted and saved in FASTQ format using the SRA Toolkit (v2.9.0) after the SRA files were gathered. The paired-end reads of fastq files were aligned, processed and iteratively corrected using HiC-Pro (v 2.11.1). Sequencing reads were mapped to genome using the bowtie2 (v2.3.4.1). Visualization of normalized Hi-C matrix and topologically associated domains was carried out by HiCExplorer (https://hicexplorer.readthedocs.io/en/latest/). All custom codes used in this study for bioinformatic analysis are available upon reasonable request from corresponding author.

For manuscripts utilizing custom algorithms or software that are central to the research but not yet described in published literature, software must be made available to editors and reviewers. We strongly encourage code deposition in a community repository (e.g. GitHub). See the Nature Research guidelines for submitting code & software for further information.

Data

Policy information about availability of data

All manuscripts must include a data availability statement. This statement should provide the following information, where applicable:

- Accession codes, unique identifiers, or web links for publicly available datasets
- A list of figures that have associated raw data
- A description of any restrictions on data availability

RNA-seq data are available in the SRA database with the accession number PRJNA532733 [https://www.ncbi.nlm.nih.gov/bioproject/PRJNA532733/] and all the 4C-seq data were deposited with GEO accession GSE155524 [https://www.ncbi.nlm.nih.gov/geo/query/acc.cgi?acc=GSE155524]. Previously published ChIP-seq data that were re-analyzed in this study are available according to the GEO accession numbers that were listed in Supplementary Table 7. Previously published Hi-C data that were re-analyzed here are available under the accession code GSE96107 [https://www.ncbi.nlm.nih.gov/geo/query/acc.cgi?acc=GSE96107]. All other data supporting the findings of this study are available on reasonable request from corresponding author.

Field-specific reporting

Please select the one below that is the best fit for your research. If you are not sure, read the appropriate sections before making your selection.

☒ Life sciences ☐ Behavioural & social sciences ☐ Ecological, evolutionary & environmental sciences

For a reference copy of the document with all sections, see nature.com/documents/nr-reporting-summary-flat.pdf

Life sciences study design

All studies must disclose on these points even when the disclosure is negative.

Sample size	The sample sizes were chosen on the basis of previous experience in accordance to the standards in the field. Experiments were performed at least 2 times to confirm reproducibility. Sample size information was described in Methods and Figure legends.
Data exclusions	Only exclusions have been made in case of failed experimental procedures based on pre-established criteria
Replication	As provided in the "Statistics and reproducibility" section in the manuscript. All the experiments for main conclusions in this study have been successfully replicated at least twice.
Randomization	Samples were all randomly allocated to different experimental groups in this study. No specific randomization protocol has been used.
Blinding	Not applicable as the extracted results are objective in this study.

Reporting for specific materials, systems and methods

We require information from authors about some types of materials, experimental systems and methods used in many studies. Here, indicate whether each material, system or method listed is relevant to your study. If you are not sure if a list item applies to your research, read the appropriate section before selecting a response.

Materials & experimental systems

n/a	Involved in the study
	☒ Antibodies
	☒ Eukaryotic cell lines
☒	Palaeontology and archaeology
	☒ Animals and other organisms
☒	Human research participants
☒	Clinical data
☒	Dual use research of concern

Methods

n/a	Involved in the study
☒	ChIP-seq
	☒ Flow cytometry
☒	MRI-based neuroimaging

Antibodies

Antibodies used

Anti-Nanog antibody (Cell Signaling, Cat No. 4893, 1:1000 for WB); Anti-Nanog antibody (Cell Signaling, Cat No. 4903, 1:100 for hESCs IF); Anti-Nanog antibody (Cell Signaling, Cat No. 8822, 1:200 for mouse embryo IF, 1:100 for ChIP-PCR); Anti-Nanog antibody (Bethyl, Cat No. A300-397A, 1:2000 for WB); Anti-Oct4 antibody (Cell Signaling, Cat No. 2750, 1:1000 for WB, 1:100 for IF); Anti-Oct4 antibody (Abcam, Cat No. ab200834, 1:1000 for WB); Anti-Sox2 antibody (Cell Signaling, Cat No. 23064, 1:1000 for WB, 1:100 for IF); Anti-Sox2 antibody (R&D, Cat No. AF2018, 1:200 for IF of mouse embryos); Anti-PHC1 antibody (Cell Signaling, Cat No.13768, 1:1000 for WB, 1:50 for IP, 1:50 for ChIP-PCR); Anti-PHC1 antibody (Active Motif, Cat No. 39723,1:200 for IF of mouse embryos, 1:100 for hESCs IF); Anti-RING1B antibody (Cell Signaling, Cat No. 5694, 1:1000 for WB, 1:200 for IP, 1:100 for hESCs IF); Anti-RING1B antibody (Abcam, Cat No. ab181140, 1:1000 for WB, 1:100 for hESCs IF); Anti-beta Actin antibody (Cell Signaling, Cat No. 3700,1:3000 for WB); Anti-beta Tubulin antibody (Abcam, Cat No. 179513, 1:3000 for WB); Anti-Ubiquityl-Histone H2A Lys119 antibody (Cell Signaling, Cat No. 8240, 1:1000 for WB, 1:100 ChIP-PCR); Anti-FLAG antibody (MBL, Cat No. M185-7, 1:3000 for WB); Anti-HA antibody (MBL, Cat No. M180-7, 1:3000 for WB); Anti-CBX7 antibody (Abcam, Cat No. ab91431, 1:1000 for WB); Anti-RYBP antibody (Abcam, Cat No. ab185971, 1:1000 for WB); Anti-Gata6 antibody (Cell Signaling, Cat No. 5851, 1:200 for IF of mouse embryos); Mouse IgG (Sigma, Cat No. I8765, 2µg for IP, 2µg for ChIP-PCR); Rabbit IgG (Cell Signaling, Cat No. 2729, 2µg for IP, 2µg for ChIP).

Validation

All antibodies used in this study are commercial and well-established in the field. Validation data are provided for each antibody on the manufacturers' websites with antibody profiles and citations: Anti-Nanog antibody (Cell Signaling, Cat No. 4893, RRID:AB_10548762, PMID:31268606); Anti-Nanog antibody (Cell Signaling, Cat No. 4903, RRID:AB_10559205, PMID:25988972); Anti-Nanog antibody (Cell Signaling, Cat No. 8822, RRID:AB_11217637, PMID:28017795, PMID:28287392); Anti-Nanog antibody (Bethyl, Cat No. A300-397A, RRID:AB_386108, PMID:33296674); Anti-Oct4 antibody (Cell Signaling, Cat No. 2750, RRID:AB_823583, PMID:33102474, PMID:31875873); Anti-Oct4 antibody (Abcam, Cat No. ab200834, RRID:N/A, PMID: 32051395); Anti-Sox2 antibody (Cell Signaling, Cat No. 23064, RRID:AB_2714146, PMID:32121397, PMID:31892848); Anti-Sox2 antibody (R&D, Cat No. AF2018, RRID:AB_355110, PMID:22700217); Anti-PHC1 antibody (Cell Signaling, Cat No.13768, RRID:AB_2716803, PMID:31029541); Anti-PHC1 antibody (Active Motif, Cat No. 39723, RRID:AB_2713961, PMID:33053359); Anti-RING1B antibody (Cell Signaling, Cat No. 5694 RRID:AB_10705604, PMID:29233865, PMID:31375680); Anti-RING1B antibody (Abcam, Cat No. ab181140, RRID:AB_2801425, PMID:31199242); Anti-beta Actin antibody (Cell Signaling, Cat No. 3700, RRID:AB_2242334, PMID:33009820); Anti-beta Tubulin antibody (Abcam, Cat NO. ab179513, RRID:N/A, PMID:31833196); Anti-Ubiquityl-Histone H2A (Lys119) antibody (Cell Signaling, Cat No. 8240, RRID:AB_10891618, PMID:31409898, PMID:32054830); Anti-FLAG antibody (MBL, Cat No. M185-7, RRID:AB_2687989, PMID:28306502); Anti-HA antibody (MBL, Cat NO. M180-7, RRID:AB_11124961, PMID:28306502); Anti-CBX7 antibody (Abcam ab91431, RRID:AB_2049271, PMID:31709304); Anti-RYBP antibody (Abcam, Cat No. ab185971, RRID:N/A, PMID:32324084); Anti Gata6 antibody (Cell Signaling, Cat No. 5851, RRID:AB_10705521, PMID:32246014); Mouse IgG (Sigma, Cat No. I8765, RRID:AB_1163672, PMID:24601879); Rabbit IgG (Cell Signaling, Cat No. 2729, RRID:AB_1031062, PMID:33230114, PMID:33088929)

Eukaryotic cell lines

Policy information about cell lines

Cell line source(s)

The list of the cell lines used in the study include H9 hESCs (WiCell), HFF (ATCC), HEK293T (ATCC), MEF cells (Innovative Cellular Therapeutics Co, Ltd, 0304-500), Mouse E14 ESCs (ATCC) and mESC line carrying the Nanog-GFP reporter (a gift from Jin Zhang Lab at Zhejiang University School of Medicine) and NCCIT(ATCC).

Authentication

Cell lines were authenticated by PCR and marker gene expression as shown in the manuscript.

Mycoplasma contamination

All the cell lines used in the manuscript were tested negative for contamination.

Commonly misidentified lines
(See ICLAC register)

None of commonly misidentified lines were used in this study.

Animals and other organisms

Policy information about studies involving animals; ARRIVE guidelines recommended for reporting animal research

Laboratory animals	For teratoma formation experiments, six-week-old male NOD/SCID mice were bred in the Experimental Animal Facility of Zhejiang University. Mice were bred up to 1 month and then subjected to histological analysis after subcutaneous injection of hESCs. For immunostaining of mouse embryos experiments, one pair of CD1 male and female mice were bred in the Center for Phenogenomics in Toronto. Immunostaining of target antibodies was performed for 20 E4.5 mouse embryos (sex was not examined). All animals were kept under specific pathogen free (SPF) and temperature-controlled environment with 12h light/12h dark cycle, and free access to food and water.
Wild animals	No wild animals were used in this study.
Field-collected samples	No field collected samples were used in the study.
Ethics oversight	As provided in the manuscript. Mice were bred in the Experimental Animal Facility of Zhejiang University. The experimental protocol and ethics were approved by the Animal Care Facility of Zhejiang University (ZJU20200034).

Note that full information on the approval of the study protocol must also be provided in the manuscript.

Flow Cytometry

Plots

Confirm that:

- [x] The axis labels state the marker and fluorochrome used (e.g. CD4-FITC).
- [x] The axis scales are clearly visible. Include numbers along axes only for bottom left plot of group (a 'group' is an analysis of identical markers).
- [x] All plots are contour plots with outliers or pseudocolor plots.
- [x] A numerical value for number of cells or percentage (with statistics) is provided.

Methodology

Sample preparation	As provided in the manuscript. Established cell line were used and no tissue processing steps were applied.
Instrument	As given in the manuscript. BD Cytomics FC 500MCL instrument was used to collect data.
Software	As provided in the manuscript. FlowJo or CXP software was used.
Cell population abundance	Not applicable. No sorting/cell purification was performed in this study.
Gating strategy	The following gating strategy was used for all the samples of relevant experiments: exclusion of cell doublets (FS Lin/AUX) and exclusion of cell debris (FS Lin/SS Lin). GFP positive cells was defined and gated using non-GFP cells as the negative control.

- [x] Tick this box to confirm that a figure exemplifying the gating strategy is provided in the Supplementary Information.
