# Supplementary material for: PHC1 maintains pluripotency by organizing genome-wide chromatin interactions of the Nanog locus
Source: Nat Commun. 2021 May 14;12:2829. doi: 10.1038/s41467-021-22871-0 (PMC8121881; doi:10.1038/s41467-021-22871-0)
Supplement: Supplementary file 4 — Description of Additional Supplementary Files [file 41467_2021_22871_MOESM4_ESM.pdf]

Descriptions of Additional Supplementary Files

Supplementary Data 1:

Description:Supplementary Table 6 of RNA seq 4 cluster

Supplementary Data 2:

Description:Supplementary Table 8 of 4C seq interactions
